# Morphology of polysorbate 80 (Tween 80) micelles in aqueous dimethyl sulfoxide solutions

**DOI:** 10.1107/S002188981000779X

**Published:** 2010-03-26

**Authors:** Hideki Aizawa

**Affiliations:** a Faculty of Pharmaceutical Sciences, Setsunan University, 573-0101, Osaka, Japan

**Keywords:** micellar structure, polysorbate 80, aqueous dimethyl sulfoxide solution

## Abstract

The structures of micelles of the surfactant polysorbate 80 (Tween 80) in 0–50% aqueous dimethyl sulfoxide (DMSO) solutions (pH 7.2, ionic strength 2.44 m*M*) were investigated by means of small-angle X-ray scattering. At DMSO concentrations of 0–20%, core–shell cylinder micelles formed, and at 30–50% DMSO, core–shell discus micelles formed, that is, changing the hydrophobicity of the DMSO solvent mixture changed the micelles from core–shell cylinder micelles to core–shell discus micelles.

## Abstract 2

A study of the structure of micelles of the surfactant polysorbate 80 in aqueous dimethyl sulfoxide solutions shows that the micelles change from core–shell cylinder micelles to core–shell discus micelles between concentrations of 20 and 30% dimethyl sulfoxide.

## 1. Introduction   

Changes in the structure of micelles of various surfactants in going from 100% water to mixtures of water and various polar solvents have attracted a lot of interest because surfactants are amphipathic, that is, they contain both a hydrophobic group and a hydrophilic group; thus they form normal micelles (hydrophobic core/hydrophilic shell micelles) in aqueous solutions, but they form reverse micelles (hydrophilic core/hydrophobic shell micelles) in organic solvents.

In recent work, we studied changes in the structure of micelles of the surfactant polysorbate 80 (Tween 80) in 0–50% aqueous polar 1,4-dioxane solutions (pH 7.2, ionic strength 2.44 m*M*) by means of small-angle X-ray scattering (SAXS). We found that polysorbate 80 micelles change from core–shell cylinder micelles to core–shell discus micelles between concentrations of 20 and 30% 1,4-dioxane, and from core–shell discus micelles to core–shell elliptic discus micelles between concentrations of 40 and 50% 1,4-dioxane (Aizawa, 2009 ▶).

Dimethyl sulfoxide (DMSO) is another polar, water-soluble organic solvent, and it is used as a solvent for Orlon and for gases such as acetylene and sulfur dioxide; as an antifreeze or a hydraulic fluid when mixed with water; and as a paint and varnish remover (Windholz *et al.*, 1976 ▶). Because its properties differ from those of 1,4-dioxane, the effects of changing the solvent from 100% water to mixtures of water and DMSO is of scientific interest. Such information can further our understanding of the underlying basic principles of micelle formation and morphology. In this study, we investigated the structure of polysorbate 80 micelles in aqueous DMSO (pH 7.2, ionic strength 2.44 m*M*) by means of SAXS, which is the best technique for determining the three-dimensional structure of micelles in solution.

## 2. Materials and method   

### 2.1. Materials and sample preparation   

Polysorbate 80 (Tween 80) and DMSO were obtained from Nacalai Tesque (Kyoto, Japan), and disodium hydrogen phosphate (Na_2_HPO_4_·12H_2_O) and sodium dihydrogen phosphate (NaH_2_PO_4_·2H_2_O) were obtained from Wako Pure Chemical Industries (Osaka, Japan). The sample preparation method is the same as that described by Aizawa (2009 ▶).

### 2.2. SAXS measurements and analysis of SAXS data   

SAXS equipment for solution analysis, including optics and a detector system, were used for this study. This equipment is installed at the Energy Accelerator Research Organization in Tsukuba, Japan, and the specifications of this equipment are detailed by Kajiwara & Hiragi (1996 ▶) and Ueki (1991 ▶). SAXS intensities were measured for 600 s for each surfactant solution and for a reference solution, and intensities were calibrated and transformed into scattering cross sections {SCS(*q*), where *q* is the scattering vector [*q* = (4π/λ)sinθ, where λ is the wavelength of the X-rays and 2θ is the scattering angle]} based on the method presented by Aizawa (2009 ▶).

The mathematical description of the core–shell cylinder model for dilute particle solutions and the least-squares fit calculation methods for the model and the SAXS data are detailed by Aizawa (2009 ▶). *n* is the number density of particles; *R* and β_core_ are the radius of the circular base of the core cylinder and the scattering length density of the core, respectively; *d* and β_shell_ are the length and the scattering length density of the shell, respectively; *H* is the height of the core–shell cylinder.

The density (1.073 g cm^−3^) of polysorbate 80 and the densities of the reference solvents were determined with a pycnometer, and the scattering length density (10.12 µÅ^−2^) of polysorbate 80 and the scattering length densities of the reference solvents were calculated from density, electron number, molecular weight, Thomson radius and the Avogadro number. The method for calculating scattering length density is detailed by Aizawa (2009 ▶). The densities and scattering length densities are listed in Table 1 ▶.

## 3. Results and discussion   

**Figure 1:**
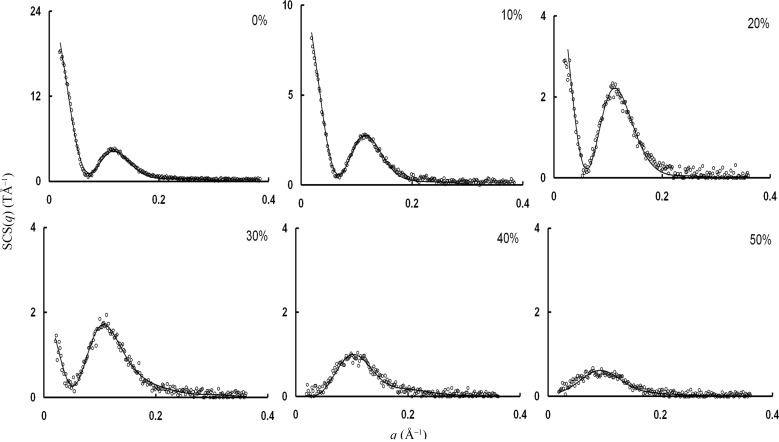
Scattering data (circles) obtained at DMSO concentrations between 0 and 50%, along with curves fitted to the core–shell cylinder model, S(*q*) = 1 (lines).

Because polysorbate 80 forms core–shell cylinder micelles in 100% water (Aizawa, 2009 ▶), we attempted to fit the SAXS data obtained at DMSO concentrations between 0 and 50% to the core–shell cylinder model for dilute particle solutions. Scattering data obtained at these concentrations are shown in Fig. 1 ▶, along with curves fitted with the core–shell cylinder model. The DMSO concentrations and shape parameters calculated from the core–shell cylinder model for dilute particle solutions are listed in Table 2 ▶. This model provided the best fit for the SAXS data at 0–50% DMSO. The SAXS data indicated that at 0–20% DMSO, polysorbate 80 formed core–shell cylinder micelles. Because (*R* + *d*) > *H* at 30–50% DMSO (see Table 2 ▶), we concluded that polysorbate 80 formed core–shell discus micelles rather than core–shell cylinder micelles at the higher DMSO concentrations. Thus, the change from core–shell cylinder micelles to core–shell discus micelles occurred at DMSO concentrations between 20 and 30%. The formation of core–shell cylinder micelles from polysorbate 80 molecules is illustrated in Fig. 3 of Aizawa (2009 ▶). At 0–20% DMSO, the polysorbate 80 molecules adopted crown-like shapes and aggregated into a ‘cylindrical’ layer of four long chains entangled with one another through intra- and intermolecular interactions. At 30–50% DMSO, core–shell discus micelles were formed by a similar mechanism.

## 4. Conclusions   

We used SAXS to investigate how changing the solvent from 100% water to a mixture of water and DMSO affected the shape and size of polysorbate 80 micelles. As the concentration of DMSO was increased, the micelles changed from core–shell cylinder micelles to core–shell discus micelles. Changing the hydrophobicity of the DMSO solvent mixture also affected the shape of the polysorbate 80 micelles. The use of other polar organic solvents with different hydrophobicities will affect the shape and size of polysorbate 80 micelles and probably change the micelles from core–shell cylinder micelles to core–shell discus micelles.

## Figures and Tables

**Table 1:** Densities and scattering length densities of the reference solvent

	DMSO concentration (%)					
	0	10	20	30	40	50
Density (gcm^3^)	0.996	1.010	1.025	1.041	1.057	1.073
Scattering length density (^2^)	9.216	9.322	9.437	9.560	9.682	9.806

**Table 2:** Shape parameters for the coreshell cylinder model [*S*(*q*) = 1] at various DMSO concentrations

Concentration (%)	*n* (a^3^)	*R* ()	*d* ()	*H* ()	_core_ (^2^)	_shell_ (^2^)
0	78.4	10	33	55	7.656	9.497
10	32.5	11	32	56	7.762	9.603
20	13.4	13	28	74	7.603	9.847
30	17.5	16	34	15	7.726	10.01
40	0.6	23	22	15	6.708	10.91
50	0.6	22	24	17	7.106	10.41
